# Expression of Ki-67 and P16 are related with HPV in squamous cell carcinoma of the external auditory canal

**DOI:** 10.1186/s40463-022-00592-8

**Published:** 2022-11-08

**Authors:** Wei Pan, Chen Zhang, Min Chen, Shiyao Min, Liang Xu, Zhangcai Chi

**Affiliations:** 1 ENT Institute and Department of Otolaryngology, Eye and ENT Hospital, Fudan University, 83 Fenyang Road, Shanghai, 200031 China; 2 NHC Key Laboratory of Hearing Medicine, Fudan University, Shanghai, 200031 People’s Republic of China; 3 Department of Pathology, Eye and ENT Hospital, Fudan University, 83 Fenyang Road, Shanghai, 200031 China

**Keywords:** Squamous cell carcinoma of external auditory canal, Ki-67, HPV, p16^INK4a^

## Abstract

**Background:**

Squamous cell carcinoma of the external auditory canal (EACSCC) is an uncommon tumor and responsible for no more than 0.2% of all the head and neck malignancies. Although there is remarkable research evidence exhibiting that high-risk human papillomavirus (HPV) accounts for considerable head and neck malignancies, its role in the pathogenesis of EACSCC is yet to be determined.

**Methods:**

We evaluated 16 patients with EACSCC treated at our department. We employed PCR to assay for high-risk subtypes of HPV. Two pathologists reviewed the histopathological staining via hematoxylin and eosin along with immunohistochemical staining of p16^INK4a^ and Ki‑67.

**Results:**

Detection of HPV DNA was done via PCR in 3 (18.75%) patients, and 8 (50%) positive (+) cases were determined via p16^INK4a^ immunostaining. Besides, 3 (37.5%) individuals were HPV positive as per p16^INK4a^ PCR results. In addition, all of the p16^INK4a^-positive specimens were diagnosed as moderately differentiated carcinomas.

**Conclusions:**

Expression of Ki-67 was related to HPV status. This is the first report implicating high-risk HPV in squamous cell carcinoma of the external auditory canal. However, p16^INK4a^ immunostaining is a suspectable approach for diagnosing HPV for EACSCC. In addition, HPV might enhance an elevated proliferation rate in EACSCC, illustrated via expression of Ki-67.

**Graphical Abstract:**

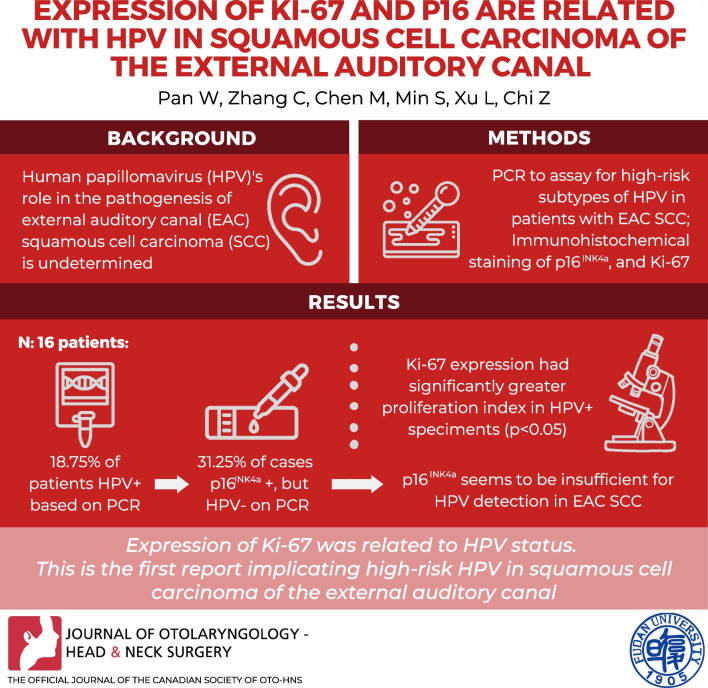

## Introduction

External auditory canal squamous cell carcinoma (EACSCC) is rare, occurring in one to six cases in about 1,000,000 persons per year [1], and is responsible for not more than 0.2% of all head and neck malignancies [2]. The global annual prevalence is approximately 1.3 cases per million persons [3]. Because of its rarity, there is no established evidence-centered treatment approach for EACSCC [4]. Based on these, it is clinically, as well as biologically remarkable to explore the molecular properties of EACSCC to assess novel treatment targets.

EACSCC, manifesting histologically as a progressive squamous dysplasia, emanates from aggregated genetic and epigenetic alterations triggered by the exposure to solar ultraviolet light and inflammatory stimuli of long-standing chronic suppurative otitis media [5]. In addition, remarkable research evidence suggests that oncogenic human papillomaviruses (HPV) genotypes 16 and 18 play an indispensable role in head and neck squamous cell carcinoma (HNSCC): infection with high-risk forms of HPV is linked to the onset of 25% of head and neck cancers [6]. However, there are no reports on the role of HPV in the carcinogenesis of the external auditory canal. Elevated rates of HPV+ tumors improved outcomes for individuals with HPV+ and enhanced survival rates in contrast with HPV− cases, which have frequently been published in HNSCC studies [7, 8]. Considering this, the diagnosis of high-risk HPV in EACSCC is necessary and useful for stratifying patients, avoiding unnecessary overtreatment and incapacitating side effects.

Infection with HPV is frequently detected via p16^INK4a^ IHC (immunohistochemistry). As a cyclin-dependent kinase repressor, the expression of p16^INK4a^ is influenced by HPV-associated oncoproteins as E7 [9, 10]. There is research evidence implicating that an inverse feedback mechanism, modulating levels of p16^INK4a^ in normal cells, gets disrupted via a decrease in the activity of pRb in growing squamous epithelial cells that are expressing HR-HPV E7 [11]. Hence, p16^INK4a^ is utilized as an HPV-linked surrogate biologic signature in numerous study methods since the procedure is well-developed and saves time [12, 13]. However, it is reported that p16^INK4a^ staining for detecting infection with HPV usually does not provide accurate results [14, 15]. Firstly, p16^INK4a^ over-expression might also be seen in tubal metaplasia along with atrophic cells and in normal columnar cells from the cervix, resulting in poor specificity [16]. Besides, p16^INK4a^ over-expression does not take place exclusively in HPV-triggered but additionally in an estimated 5–10% of HPV-negative HNSCC [13, 17, 18]. Moreover, numerous trends of p16^INK4^ expression result in diverse interpretations of "p16^INK4a^-positivity" [19–21]. Thus, this situation restricts the utilization of p16^INK4a^ for diagnosing HPV infection in HNSCC.

Ki-67 constitutes a nuclear antigen as well as a biological signature of cellular proliferation, which is expressed in all stages of the cell cycle except G0 [22]. p16 and Ki-67 expression are mutually exclusive in normal cells. Recently, the simultaneous detection of p16^INK4a^ and Ki-67 in a cell implies epithelial cell transformation that might progress to cancer [23]. p16^INK4a^ has two diametrically opposed biological roles in cell cycle regulation. Conversely, p16^INK4a^ expression could serve as a pivotal modulator of cellular senescence [24]. On the contrary, p16^INK4a^ expression can be triggered upon signaling of HPV E7 oncogene [25]. Thus, p16INK4a /Ki-67 dual staining is useful to differentiate successfully cell cycle arrest from malignantly transformed p16^INK4a^-positive cells.

The objectives of this research work were twofold: first, to investigate the incidence of HPV in EACSCC; second, to explore the clinical performance of p16^INK4a^ /Ki-67 as a marker for detecting HPV-induced transformed cells in EACSCC.

## Materials and methods

### Patients and specimens

Sixteen individuals with EACSCC were enrolled in this research work, with a mean age of 57 years, ranging from 1 to 76 years, with a male/female ratio of 10:6. The histopathologic assessment of all samples was confirmed and verified by two board-certified pathologists. The research work was granted approval by the Institutional Ethics and Review Committee of the Faculty of Clinical Medicine, Eye and ENT Hospital, Fudan University, Shanghai, China.

### Immunohistochemistry

Immunohistochemical analysis was done on five μm-thick paraffin sections of EACSCC and adjacent normal skin tissue. The following deparaffinization with xylene, and subsequent rehydration via 96% ethanol, blocking of the activity of endogenous peroxides was done with 3% hydrogen peroxide for 10 min and rinsed in PBS. Exposing of the antigens was done by boiling the sections in citrate buffer (pH 6.0) for 15 min. After that, the primary antibody, anti-p16^INK4a^ anti-Ki67, was introduced overnight. The slides were inoculated for 10 min with secondary antibody (Biotinylated Goat Anti-Polyvalent, NeoMarker, United States). The immunoperoxidase labeling was done; diaminobenzidine (DAB, Zymed, United States) was utilized as a chromogen for visualizing docking of the antibody. Finally, we stained the sections with Harris's haematoxylin, followed by clearing and mounting.

For p16^INK4a^, the presence of nuclear staining with or without cytoplasmic staining was regarded as a positive result. The tumoral p16^INK4a^ expression trends were documented and categorized as per following criteria: (1) diffuse (> 10% of tumor cells harboring p16^INK4a^ expression exhibiting clonal formation trend); (2) focal (numerous p16^INK4a^-positive cells not harboring criteria for diffuse p16^INK4a^ expression).

Ki‑67 expression was stratified into four groups as per the distribution along with the fractions of cells with positive nuclear staining as shown: Score 0, < 10% of the cells; score 1, 10‑29% of the cells; score 2, 30‑69% of the cells; score 3, ≥ 70% epithelial cells [26].

### Detection and genotyping of human papillomavirus DNA

Total genomic DNA was extracted from fresh frozen tissue of all samples using the TIANamp Genomic DNA Kit (Tiangen Biotech, Beijing, China). HPV detection and genotyping were done using the Human Papillomavirus (HPV) Genotyping Real-Time PCR Kit (Liferiver Bio-Tech, CA, United States). The Luminex bead-based genotyping approach allows for the diagnosis of 15 high-risk forms (16, 18, 31, 33, 35, 39, 45, 51, 52, 56, 58, 59, 68, 73, and 82) and six low-risk types (6, 11, 42, 43, 44, and 70), according to the manufacturer’s instructions. The HPV genotypes were categorized as HR‑HPV or LR‑HPV based on the scheme opined by Dunne et al.

### Statistical analysis

SPSS software (version 22.0; SPSS, Inc., Chicago, IL, USA) was used for statistical analysis. Fischer's tests and non-parametric Mann–Whitney's test were used for univariate analysis. P < 0.05 was considered to indicate a statistically significant difference (Table 1).

## Results

### HPV DNA status

3 (18.75%) patients were HPV + based on PCR results (Table 2). Only in 3 (18.75%) cases did the PCR result match the positive p16^INK4a^ staining (Table 2). In 5 (31.25%) cases, p16^INK4a^ staining was positive, but the PCR result was negative (Table 2). In summary, p16^INK4a^ staining for diagnosing HPV is inadequately sensitive and specific.

Among the 3 HPV positive specimens, the most often diagnosed HPV type was HPV-16 (66.7%), followed by HPV-18 (33.3%). Low-risk type HPV DNA (HPV-6/11) was not found in all specimens.

### Expression of p16^INK4a^ and Ki-67 in EACSCC

**Fig. 1:**
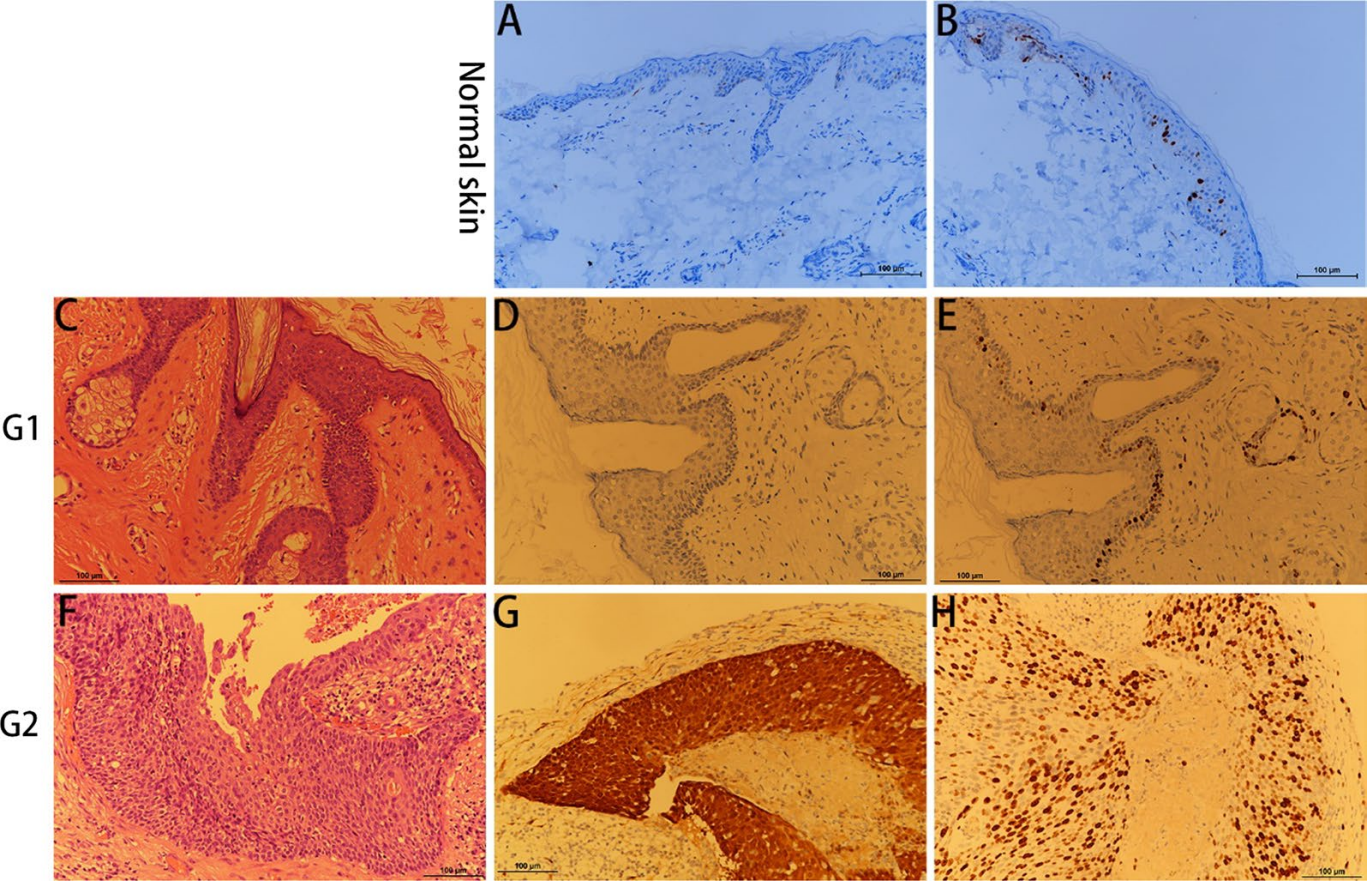
p16^INK4a^ and Ki-67 expression in normal skin (**A** p16^INK4a^; **B** Ki-67), well differentiated squamous cell carcinoma (G1; **C** H&E stain; **D** p16^INK4a^; **E** Ki-67) and moderately differentiated squamous cell carcinoma (G2; **F** H&E stain; **G** p16^INK4a^, **H** Ki-67)

Complete p16^INK4a^ expression absence was seen in 8 of 16 (50%) EACSCC (Table 1). The remaining EACSCC were scored with varying extent as well as the pattern of p16^INK4a^ expression (Fig. 1 and Table 1). As demonstrated in Table 1, a diffuse p16^INK4a^ expression pattern was frequently observed in EACSCC. The 16 EACSCC were categorized as well to moderately differentiated (Fig. 1) as follows: G 1 (eight cases), G 2 (eight cases). It is pivotal to mention that all eight moderately-differentiated (G 2) EACSCC displayed p16^INK4a^ positivity (Table 3). Comparing the well (G1) with moderately differentiated (G2) EACSCC, it was noted that all cases diagnosed as G2 presented more frequent p16^INK4a^ positivity, whereas the G1 cases were p16^INK4a^ negative (Table 3).

**Table 1:** p16^INK4a^ and Ki-67 evaluation parameters in EACSCC

Evaluation parameter	EACSCC Sample number
*p16*^*INK4a*^* intratumoral distribution*	
Negative	8/16 (50%)
Focal	2/16 (12.5%)
Diffuse	6/16 (37.5%)
*Ki-67 staining intensity*	
0	4/16 (25%)
1	5/16 (31.25%)
2	6/16 (37.5%)
3	1/16 (6.25%)

**Table 2:** Comparison of p16^INK4a^, Ki-67 result and final diagnosis according to HPV infection status

HPV status	p16^INK4a^		Ki-67 score
	+	−	
High-risk (n = 3)	3	0	7
Negative (n = 13)	5	8	13

**Table 3:** Clinicopathological and p16INK4a of EACSCC upon the histological grade of differentiation

Parameter	G1 (n = 8)	G2 (n = 8)	P-value*
p16^INK4a^-positive cases	0 (0%)	8 (100%)	< 0.05

G1, Well differentiated; G2, Moderately differentiated

*Two-sided Fisher's exact test

Ki-67 expression was observed in 15 of 16 (93.7%) EACSCC (Table 1). The greater proliferation index was documented in HPV-positive specimens with 2.34 mean PI value, which was 1.0 in HPV-negative specimens (Fig. 1 and Table 2). The difference between groups was significant (p = 0.0383; p < 0.05). However, there was no remarkable difference in Ki-67 expression scores between the well (G1) and moderately differentiated (G2) of EACSCC. Furthermore, concomitant with diffuseness, a high Ki‑67 index was implicated in the HR‑HPV group.

## Discussion

To investigate the presence of HPV DNA, we studied 16 cases of surgically removed primary SCC in the external auditory canal. We carried out a dual investigation: a molecular biology study (using PCR to diagnose the presence of HPV DNA) and immunohistochemical profiling (to investigate the expression of p16^INK4a^ protein). Furthermore, 3 of 16 patients were found to have detectable HPV 16 and HPV 18 DNA. Our data align with the other research conducted by Masterson et al. [1]. Although the 18.75% detection rate of high-risk HPV-DNA in this research work represents a minority, the clinical significance may be apparent that high-risk HPV is implicated in squamous cell carcinoma of the external auditory canal. However, a potential limitation may be the lack of an adequate sample size because of its rarity.

It is reported that p16^INK4a^ was an insensitive signature of HPV condition with a low estimation significance for HPV infection [27]. This supports that p16^INK4a^ staining might be an inadequate HPV detection approach because we reported a high rate of failure in cases that were positive for p16^INK4a^ but were actually HPV−. Through this method, the number of false-positive HPV+ cases would be 50 percent higher. Presently, ultraviolet along with ionizing radiation constitutes the only evidence-centered supported risk factors for TB SCC [28]. In addition, the previous investigations illustrated that the ultraviolet B irradiation could activate the CDKN2A (cyclin-dependent kinase inhibitor 2A) gene that encodes the p16^INK4a^ protein in human keratinocytes [29–32]. On the other hand, activation of CDKN2A in keratinocytes seems to be related to tumor de-differentiation in head and neck areas [33]. In our study, activation of p16^INK4a^ protein was exceeded in one-half of the EACSCCs. Notably, a strong relationship between p16^INK4a^ over-expression and the degree of tumor differentiation is seen. These facts suggest that p16^INK4a^ should be necessary to induce tumor progression and de-differentiation.

Ki‑67 constitutes a nuclear protein that is linked to RNA transcription along with the progress of cell cycle [11]. The present research work explored the efficacy of Ki‑67 in the EACSCC and compared the findings of HR‑HPV and HPV‑negative groups. The assessment of the data shows a considerable relationship between the detection of the HPV genome and the immune-histochemical expression of Ki-67. Hence, it appears that HPV can be an indispensable cofactor in the onset and progression of squamous carcinoma of EAC. In squamous carcinoma, HPV appears to increase direct changes in the mitosis along with multiplication phases of the cell cycle that could be estimated via the expression of Ki-67, triggering a higher cell proliferation index. Thus, HPV may increase the proliferation index in EACSCC, exhibited via the expression of Ki-67.

In summary, our data show that high-risk HPV 16 and HPV 18 are related to the SCC of EAC. p16^INK4a^ immunostaining seems to be insufficient for HPV detection in EACSCC. However, a p16-dependent cascade might be implicated in tumor de-differentiation of EACSCC. Furthermore, we found an impressive link of the presence of HPV to Ki-67 expression. Because the number of patients in our study was limited, a larger study is necessary to address this issue.

## Data Availability

All data generated or analyzed during this study are included in this published article.

## Abbreviations

EACSCC: Squamous cell carcinoma of the external auditory canal
HPV: Human papillomavirus
PCR: Polymerase chain reaction
IHC: Immunohistochemistry
HNSCC: Head and neck squamous cell carcinoma
PBS: Phosphate buffered saline
HR-HPV: High-risk human papillomavirus
LR-HPV: Low-risk human papillomavirus
TBSCC: Temporal bone squamous cell carcinoma
CDKN2A: Cyclin-dependent kinase inhibitor 2A
